# Temperature modulates gut microbiome disruption and resistome enrichment in oxytetracycline-treated channel catfish (*Ictalurus punctatus*)

**DOI:** 10.1128/spectrum.04187-25

**Published:** 2026-03-30

**Authors:** Xiran Li, Hongye Wang, Hisham A. Abdelrahman, Anita M. Kelly, Luke A. Roy, Esteban Soto, Luxin Wang

**Affiliations:** 1 Department of Food Science and Technology, University of California Davis 117239, Davis, California, USA; 2 Department of Biological Sciences, California State University East Bay 14667, Hayward, California, USA; 3 Department of Veterinary Hygiene and Management, Faculty of Veterinary Medicine, Cairo University 110151 , Giza, Egypt; 4 Department of Biology, Marine Biology, and Environmental Science, Roger Williams University 170324, Bristol, Rhode Island, USA; 5 Alabama Fish Farming Center 115417 , Greensboro, Alabama, USA; 6 School of Fisheries, Aquaculture & Aquatic Sciences, Auburn University 218456, Auburn, Alabama, USA; 7 Department of Medicine and Epidemiology, School of Veterinary Medicine, University of California Davis 70733, Davis, California, USA; University of Guelph College of Biological Science, Guelph, Canada

**Keywords:** gut microbiome, catfish, oxytetracycline, antimicrobial resistance, aquaculture

## Abstract

Oxytetracycline (OTC) is one of the few antibiotics approved by the U.S. Food and Drug Administration for catfish aquaculture. Unfortunately, OTC resistance has been frequently detected in production environments, with the fish gut identified as a potential hotspot for resistance selection. In aquaculture systems, water temperature is a critical factor influencing fish physiology, antibiotic pharmacokinetics, and water resistome development. However, its role in modulating OTC effects on the fish gut microbiome remains underexplored. This study examined temperature-dependent microbiome and resistome responses in channel catfish (*Ictalurus punctatus*) when treated with OTC at 20°C, 25°C, and 30°C. Gut contents collected at treatment completion and after withdrawal were analyzed via metagenomic sequencing. In untreated fish, temperature alone shaped microbial structure and function, with the Shannon diversity increasing with temperatures and the β-diversity differing significantly across temperature groups. After OTC exposure, microbial responses were markedly temperature dependent with few taxa affected at 20°C, whereas substantial shifts occurred at 25°C and 30°C, indicating reduced microbial resilience at higher temperatures. OTC elevated total antimicrobial resistance gene (ARG) abundance, enriching tetracycline and β-lactam resistant genes consistent with co-selection. ARG–host linkages were diffuse at 20°C but consolidated within *Klebsiella*, *Enterococcus*, *Enterobacter*, and *Paraclostridium* at 25°C and 30°C. Notably, OTC-induced dysbiosis persisted through the withdrawal period. These findings demonstrate that temperature modulates both the magnitude and persistence of OTC-driven microbiome disruption and resistome enrichment, underscoring the importance of temperature-aware antibiotic management to mitigate antimicrobial resistance risks and safeguard fish health and food safety in aquaculture.

**IMPORTANCE:**

This study reveals that water temperature critically shapes how antibiotics affect the gut microbiome and antimicrobial resistance in channel catfish. Metagenomic sequencing results showed that oxytetracycline (OTC) treatment caused minimal disruption of the microbiome at 20°C, but induced significant community shifts and enrichment of antimicrobial resistance genes (ARGs) at 25°C and 30°C. Higher temperatures reduced microbial resilience, consolidating ARGs within key bacterial genera such as *Klebsiella* and *Enterococcus*. Importantly, OTC-induced microbiome changes and resistance persisted through the withdrawal period. These findings highlight temperature as a major driver of antibiotic impact in aquaculture, emphasizing the prudent use of antibiotics at different disease breakout temperatures.

## INTRODUCTION

Since capture fisheries plateaued in the 1980s, global fish production growth has been driven primarily by aquaculture to meet increasing demand (1). In 2022, aquaculture production surpassed capture fisheries for the first time, accounting for 51% of total aquatic animals destined for human consumption (1). This expansion has been enabled by a shift from extensive to intensive farming practices (2). In the United States, catfish are the leading freshwater culture species, with an annual production of 329 million pounds (3). However, intensified production increases the risk of disease outbreaks caused by viral, bacterial, fungal, and parasitic pathogens, leading to greater reliance on chemicals and antimicrobials (4).

Oxytetracycline (OTC) is a naturally derived, broad-spectrum antibiotic. In the United States, the dihydrate form of OTC is approved for use in catfish aquaculture, where it is administered via medicated feed during disease outbreaks to control bacterial hemorrhagic septicemia caused by *Aeromonas hydrophila* and *Pseudomonas* disease caused by *Pseudomonas* spp. (5). However, OTC resistance is now found in various aquaculture settings. OTC resistance genes (6–8) and OTC-resistant bacteria have been frequently detected in aquaculture systems and surrounding environments (6, 9). For example, 75%–82.5 % of 260 *Aeromonas* spp. collected from diseased and healthy African catfish on 36 Nigerian earthen-pond farms were highly resistant to OTC (9). Similarly, 50% of 31 *Vibrio* spp. from Brazilian shrimp farm water and 20% of 94 *Edwardsiella tarda* isolates from diseased eels exhibited OTC resistance (10, 11). In addition, *Pseudomonas*, *Acinetobacter*, and *Moraxella* spp. have been found to be frequent carriers of OTC resistance genes such as *tet(M)*, *tet(B)*, *tet(A)*, *and tet(34)* (12–15). The occurrence of OTC-resistant bacteria in aquaculture environments not only compromises the efficacy of antibiotic treatments but also poses significant ecological and public health risks. The selection of increasingly resistant bacterial strains can lead to more frequent and severe disease outbreaks, resulting in substantial economic losses (16, 17). Moreover, resistant microorganisms may enter the human food chain through consumption of raw or undercooked contaminated fish products, representing an indirect yet concerning route of exposure (18).

The origin and transmission dynamics of OTC resistance genes in aquaculture systems remain poorly understood. Some studies suggest that resistance selection may occur primarily within the fish intestine rather than in surrounding sediments. For example, Muziasari et al. (19) proposed that fish feces serve as a major contributor to the accumulation of antimicrobial resistance genes (ARGs) in sediments and identified the gut as a potential hotspot for both ARGs and mobile genetic elements (MGEs), as well as a likely source of their dissemination. Wang et al. (20) observed that florfenicol treatment induced more pronounced shifts in the catfish intestinal microbial diversity than in gill and skin communities, underscoring the gut’s heightened sensitivity and potential role in ARG selection. While ARGs and MGEs have been studied in fish feces (19), fishmeal (21), and sediments (22), the fish gut microbiome itself remains an underexplored niche.

Catfish are typically raised in shallow earthen ponds where seasonal weather conditions closely influence water temperature (23–26). Due to minimal thermal stratification, pond water temperatures closely correlate with ambient air temperatures (27, 28). As ectotherms, fish have body and gut temperatures that mirror their surrounding environment (29). Water temperature significantly influences fish physiology, health, body composition, and gut microbiota (29, 30), and the pharmacokinetics of OTC in fish (31). Additionally, it affects antibiotic effectiveness, shaping the aquatic resistome, as well as the abundance and composition of antibiotic-resistant bacteria in aquaculture systems (8). Therefore, we hypothesized that water temperature modulates the impact of OTC on the channel catfish (*Ictalurus punctatus*) gut microbiome and antimicrobial resistance (AMR) development. To test this, our study examined the effects of OTC on microbial population dynamics and resistome profiles in the gut of channel catfish at the end of OTC administration and the withdrawal period under three commercially relevant water temperatures, 20°C, 25°C, and 30°C, representing the typical range for bacterial disease outbreaks in aquaculture (8).

## MATERIALS AND METHODS

### Catfish trial setup

Twenty-four 132 L culture tanks were set up for the trial at the Alabama Fish Farming Center in Greensboro, AL. Each tank was filled with water sourced from a surface watershed reservoir and equipped with an individual biofilter, which created an independent recirculating system for each tank. The experiment involved three temperature treatments (20°C, 25°C, and 30°C), each replicated across four tanks. To stimulate the colonization of nitrifying bacteria on the biofilters, ammonium chloride (Fritz PRO, Mesquite, TX) was added to each tank 3 weeks prior to the pre-conditioning phase, following the manufacturer’s instructions. Throughout the study, dissolved oxygen levels were maintained between 6.4 and 7.0 ppm. Two weeks before the experiment began, channel catfish fingerlings (average weight: 18.7 ± 6.45 g) were stocked at a density of 25 fish per tank and gradually acclimated to their respective temperature conditions during the pre-conditioning phase. During this period, catfish were fed a standard control diet (basal feed) containing 32% crude protein and 6% total lipid at approximately 2.5% of their body weight per day. During the treatment phase, fish in the control (CON) group continued receiving the control diet, while those in the treatment groups (OTC) were fed a Terramycin-medicated diet. The medicated feed was prepared by thoroughly mixing 13.228 g of Terramycin 200 (44.1% active oxytetracycline, Phibro Animal Health) with 2,986.77 g of basal feed, yielding a final pre-mix concentration of 0.4409% (equivalent to 0.195% active oxytetracycline). The mixture was homogenized for 15 minutes to ensure even distribution of the antibiotic. Prior to feeding, the pellets were manually broken into smaller pieces to suit the size of the fingerlings. Feed was administered gradually to feed was administered for 10 consecutive days, followed by a 21-day drug withdrawal period during which all fish were switched back to the basal feeds. Throughout the experimental period, water quality was consistently assessed. The mean temperatures for the 20°C, 25°C, and 30°C treatments were stabilized at 20.3 ± 0.21°C, 25.4 ± 0.24°C, and 30.2 ± 0.08°C, respectively. Measured pH values ranged between 6.18 and 7.38, and total ammonia nitrogen levels fluctuated from 0.007 to 7.2 ppm. To safeguard fish health, un-ionized ammonia levels were consistently kept below 0.1 mg/L, in accordance with safety guidelines outlined by Zhou and Boyd (32) and Colt and Tchobanoglous (33).

### Catfish intestinal content sampling, DNA extraction, and sequencing

Three catfish were taken from each tank at three sampling points, including at the end of the pre-conditioning period (prior to treatment), at the conclusion of the treatment period, and at the end of the withdrawal phase. Fish were euthanized using a tricaine methanesulfonate (MS-222) solution (500 mg/L; TCI America, Portland, OR), buffered with sodium bicarbonate (Arm & Hammer, Trenton, NJ) (34). Due to the small size of fish fingerlings, the entire content of every intestine was collected using FLOQSwabs (Copan Diagnostics, Murrieta, CA). The swab was then placed into a PowerBead Pro tube from the DNeasy PowerSoil Pro Kit (QIAGEN, Valencia, CA) and stored at −20°C until DNA extraction.

DNA was extracted from the swabs using the DNeasy PowerSoil Pro Kit, following the manufacturer’s instructions. Due to the low microbial load in the fish intestinal samples, DNA from fish within the same tank was pooled for library preparation and sequencing. The purity and concentration of the extracted DNA were initially assessed using a NanoDrop spectrophotometer 2000c (NanoDrop Technologies, DE, USA) by measuring absorbance at 230, 260, and 280 nm. Additional quantification was performed using a Qubit Fluorometer (Life Technologies, Carlsbad, CA). Sequencing libraries were prepared with the QIAseq FX DNA Library Kit (QIAGEN), and the library quality and concentrations were evaluated using a Bioanalyzer 2100 (Agilent, Santa Clara, CA). High-throughput sequencing was performed at the UC Davis Genome Center using the Illumina NovaSeq 6000 platform, generating 150 bp paired-end reads.

### Quality filtering, contig assembly, and general bioinformatic analysis

Raw sequencing reads underwent initial quality control using Trimmomatic (v0.39) (35) with default parameters, including removal of Illumina adapters, sliding–window trimming (four bases, average quality ≥15), and discarding reads shorter than 36 bp. To eliminate host-derived sequences, reads aligning to the catfish reference genome (NC_030416.1) were removed using Bowtie 2 (v2.5.3) (36). After host filtering, samples contained 3,236,111–103,143,722 reads. Taxonomic classification of quality-filtered, host-depleted reads was performed using Kraken2 (v2.1.5) with the standard Kraken database (RefSeq bacteria, archaea, and viral genomes; the GRCh38 human genome; and UniVec_Core vectors) and default parameters (k-mer = 35) (37). Relative abundances were subsequently estimated at the genus level using Bracken (v2.7.0) (38). ANCOM-BC2 (39) was applied to identify significantly different taxa between treatments under each temperature condition.

*De novo* metagenomic assembly was carried out independently for each sample using MEGAHIT (v1.2.9) with default settings (40). Functional annotation was performed using eggNOG-mapper (v2) (41). Briefly, open reading frames were predicted from assembled contigs using Prodigal (v2.6.3) in metagenomic mode (-p meta) (42). Predicted proteins were aligned to the eggNOG (v5) database (43) using DIAMOND (v2.1.11) (44) in “–more-sensitive” mode. The resulting orthologous groups were then mapped to the Clusters of orthologous groups (COG) database (45) for functional annotation, and the best hits were retained. Annotation counts were normalized by the total number of annotated hits to account for sequencing depth and enable cross-sample comparisons. For functional enrichment, a likelihood ratio test (LRT) implemented in DESeq2 (46) was used to compare full and reduced models. Orthologous groups with LRT *P* < 0.05 were used to generate ternary plots. Statistical differences in total ARG abundance were assessed by testing normality with the Shapiro–Wilk test, followed by one-way ANOVA and Tukey’s post hoc test.

### Prediction and quantification of antimicrobial resistance genes (ARGs) and their bacterial hosts

ARG analysis was performed using ResFinder (v2.5.0) (47) on MEGAHIT-assembled contigs, focusing on acquired ARGs. To account for biases introduced by differences in ARG reference sequence length, ARG abundance in read-based analyses was normalized by both the reference gene length and sequencing depth and expressed as “ARGs per copy of 16S rRNA gene” following Liu et al. (48):


$${ARG}_{i}\;abundance=\frac{\sum_{1}^{n}N_{ARG\;like\;sequence}\;\times \;\left(L_{reads}/L_{ARG\;reference\;seqence}\right)}{\left(N_{16S\;sequence}\;\times L_{reads}/L_{16S\;sequence\;}\right)},$$

where *n* denotes each individual ARG; $N_{ARG\;like\;sequence}$ is the number of reads mapped to the ResFinder database; $L_{reads}$ is the read length; $L_{ARG\;reference\;seqence}$ is the length of the corresponding ARG reference sequence in the database; $N_{16S\;sequence}$ is the number of 16S rRNA sequences identified per sample using METAXA2 (v2.1.3) (49); and $L_{16S\;sequence\;}$ is the average length of a 16S rRNA gene (1,432 bp, based on the Greengenes database).

ARG-containing contigs were subsequently extracted by first obtaining contig identifiers associated with resistance genes from the ResFinder output tables (ResFinder_results_tab.txt) using awk and storing them as sample-specific text files. These identifiers were then used with SeqKit (v2.6.1) (50) to retrieve the corresponding sequences from the assembly FASTA files (*.contigs.fa). ARG-associated contigs were compiled into sample-level FASTA files and filtered using SeqKit to remove sequences shorter than 500 bp. Taxonomic assignment was then performed using Kraken2 with the standard database. The resulting classification reports were used to analyze ARG–host associations.

### Statistical analysis

Taxonomic profiles generated using Bracken were processed in R (v4.2.2) with the tidyverse (v1.3.2) (51) and phyloseq (v3.18) (52) packages. Bracken reports from all samples were imported and combined into a single table, with reads classified as *Homo sapiens* removed. Bracken was run at two taxonomic resolutions. For diversity and multivariate analyses, Bracken estimated counts were generated at the finest available taxonomic level (species level) and used to construct the abundance matrix. For taxonomic composition visualization, Bracken was run again with outputs summarized at the genus level, and genus-level estimated counts (new_est_reads) were used for composition plots. Taxonomic information provided by Bracken (taxon names and IDs) was reformatted into a standard hierarchical taxonomy table. The abundance matrix, taxonomy, and sample metadata were integrated into a unified phyloseq object.

α-Diversity was evaluated using the Shannon diversity index within the phyloseq R package. Data normality was assessed using the Shapiro–Wilk test (*stats* v3.6.2). As the data did not follow a normal distribution, differences in Shannon diversity across temperature treatments were analyzed using the non-parametric Kruskal–Wallis test (53), followed by Dunn’s post hoc test (*dunn.test* v1.3.5) for pairwise comparisons (54).

β-Diversity was assessed using Bray–Curtis dissimilarity matrices. Differences in community structure across temperature treatments were visualized using principal coordinate analysis, and statistical significance was tested using permutational multivariate analysis of variance (PERMANOVA) with 1,000 permutations.

To quantify treatment-induced community shifts, Bray–Curtis dissimilarities were calculated between each sample and the centroid of the corresponding control group at the same temperature. The centroid was defined as the multivariate average (mean relative abundance of OTUs) of all control samples in the Bray–Curtis space. Each sample’s distance to this centroid served as a measure of deviation from baseline community structure. Differences in distance-to-centroid values across treatments were evaluated using the Kruskal–Wallis test, with pairwise Wilcoxon rank-sum tests and Benjamini–Hochberg correction applied for multiple comparisons. Effect sizes (*r*) from Wilcoxon tests were calculated to quantify the magnitude of differences, interpreted as small (*r* = 0.1), moderate (*r* = 0.3), or large (*r* ≥ 0.5).

## RESULTS

**Fig 1:**
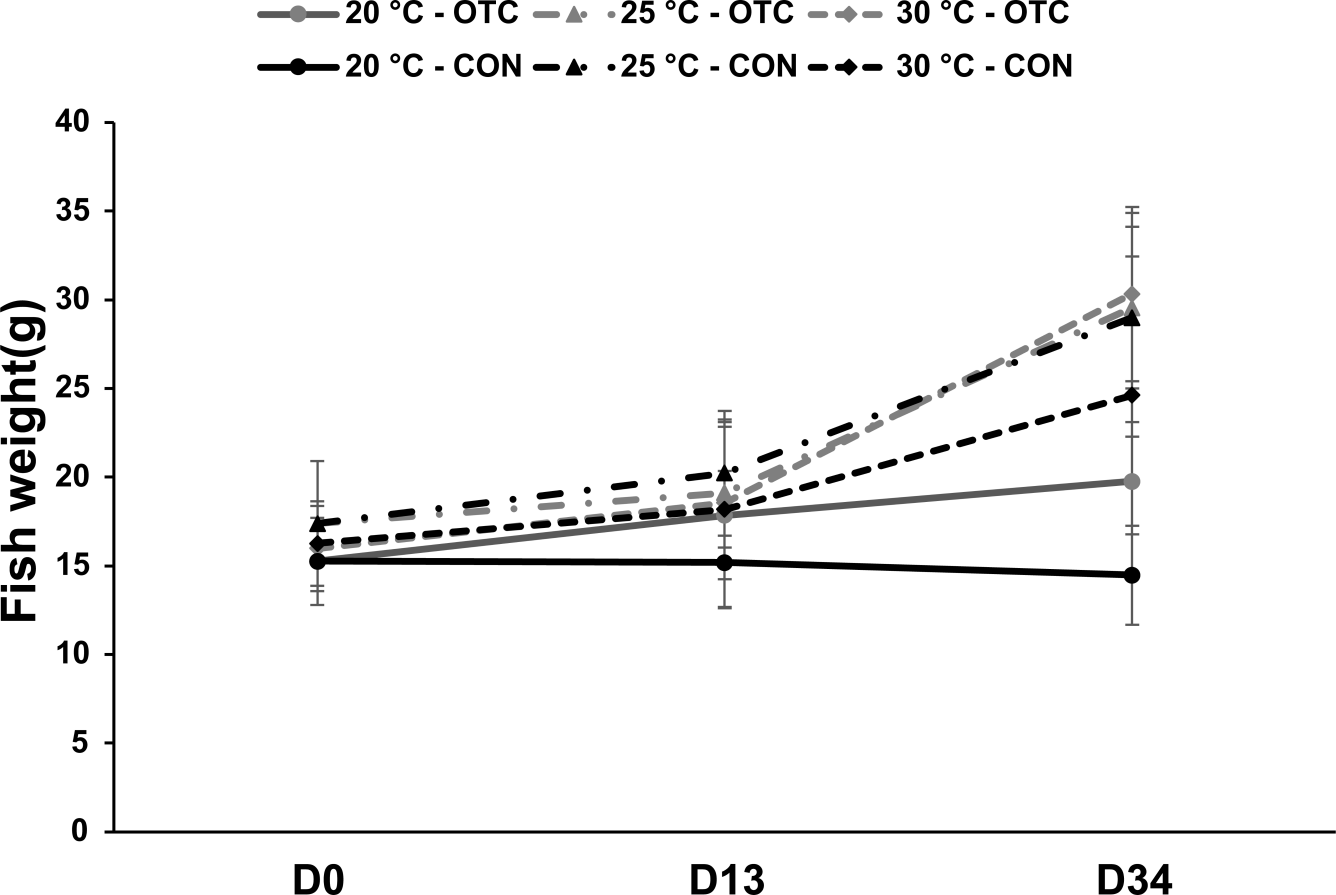
Variation in fish weight of channel catfish (*Ictalurus punctatus*) fingerlings in control (CON) and oxytetracycline (OTC)-treated tanks before treatment, after treatment, and at the end of the withdrawal period.

In the OTC-treated group, fish weight increased over the experimental period from 19.8 ± 2.51 g at 20°C to 29.6 ± 4.57 g at 25°C and 30.3 ± 4.92 g at 30°C. In the control group, final weights reached 15.5 ± 2.78 g at 20°C, 29.0 ± 5.91 g at 25°C, and 24.6 ± 7.8 g at 30°C (Fig. 1). Overall, no significant effect of OTC treatment on weight gain was observed (*P* > 0.05).

### Temperature shapes structural and functional baselines of the fish gut microbiome

**Fig 2:**
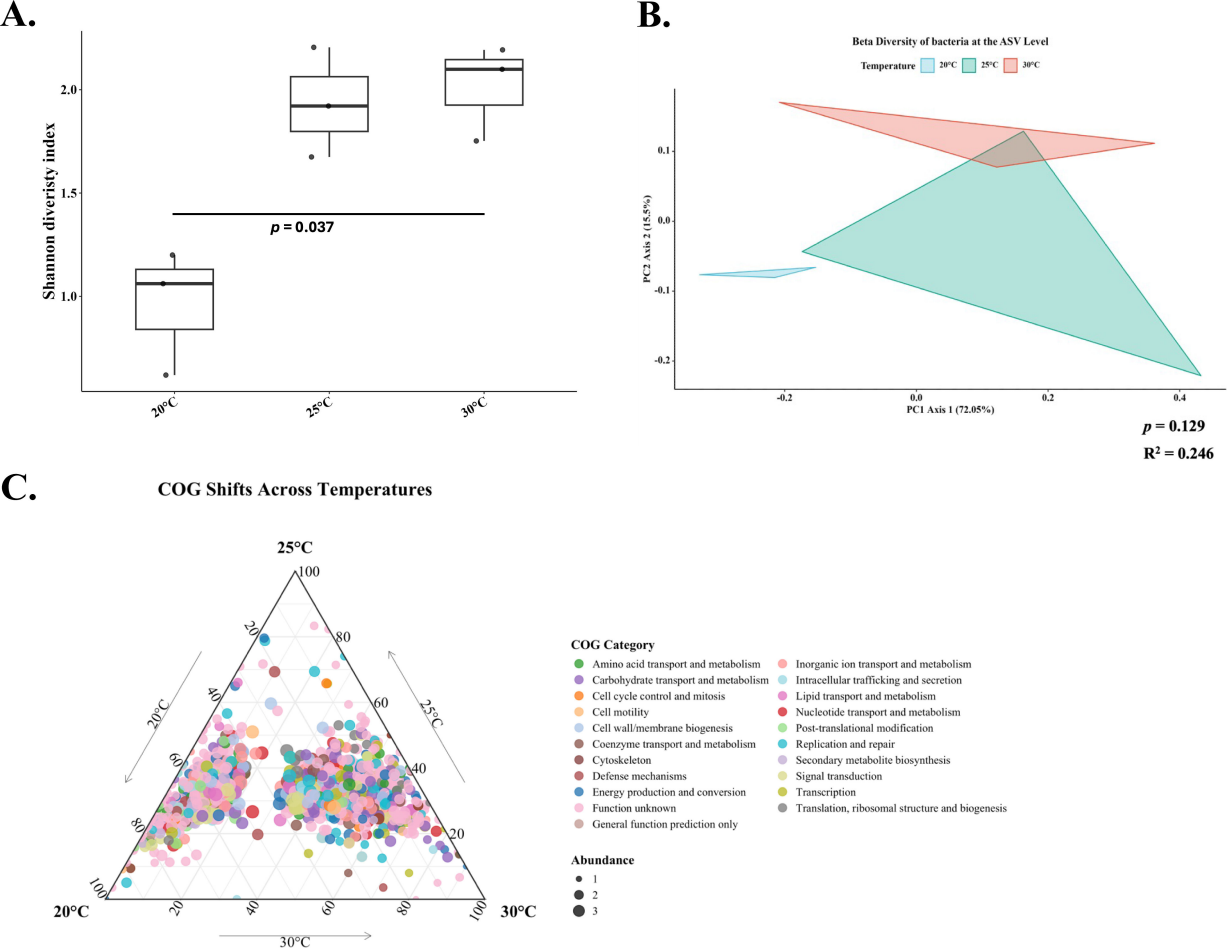
Baseline microbial community (pre-treatment) shifts in the gut of channel catfish (*Ictalurus punctatus*) fingerlings under different water temperatures prior to antibiotic treatment. (**A**) Shannon diversity indices of the catfish gut microbiome. *P* values were obtained using a Kruskal–Wallis test followed by Dunn’s post hoc test for pairwise comparisons (adjusted *P* values). Only significant comparisons (*P* < 0.05) are indicated. (**B**) Principal coordinate analysis based on Bray–Curtis dissimilarity showing differences in gut community composition (β-diversity) among temperatures. Group differences were tested using PERMANOVA (adonis2); the *R*² value (variance explained) and adjusted *P* value are reported on the plot. (**C**) Ternary plot showing shifts in predicted bacterial functional profiles, highlighting genes significantly enriched under different temperatures. Significance was determined using the likelihood ratio test in DESeq2 (*P* < 0.05). Dot color represents the associated Clusters of Orthologous Groups (COG) category; dot size corresponds to gene abundance, plotted on a logarithmic scale.

In the absence of OTC exposure, water temperature exerted a clear influence on the gut microbiome of channel catfish. Microbial diversity increased with rising temperature, as the Shannon diversity index at 30°C was significantly higher than at 20°C (*P* = 0.037; Fig. 2A). β-Diversity analysis showed an apparent temperature-associated pattern in community composition (Fig. 2B). However, PERMANOVA did not detect a statistically significant difference among temperatures (*P* > 0.05). The R² value (0.246) suggests that temperature accounted for ~24% of the variance in Bray–Curtis dissimilarities, although this effect was not statistically supported in our data set. These compositional shifts were accompanied by significant functional changes (Fig. 2C). An LRT identified 1,184 COGs significantly affected by temperature (*P* < 0.05; Fig. 2C). Of these, 29.0% were associated with unknown functions, 9.5% with energy production and conversion, 7.9% with carbohydrate transport and metabolism, and 5.83% with replication and repair.

Together, these findings demonstrate that temperature shapes both the structure and function of the catfish gut microbiome. Accordingly, subsequent analyses of OTC impact were stratified by temperature to account for these distinct microbial community baselines.

### Temperature-dependent shifts in gut microbial composition and diversity under antibiotic pressure

**Fig 3:**
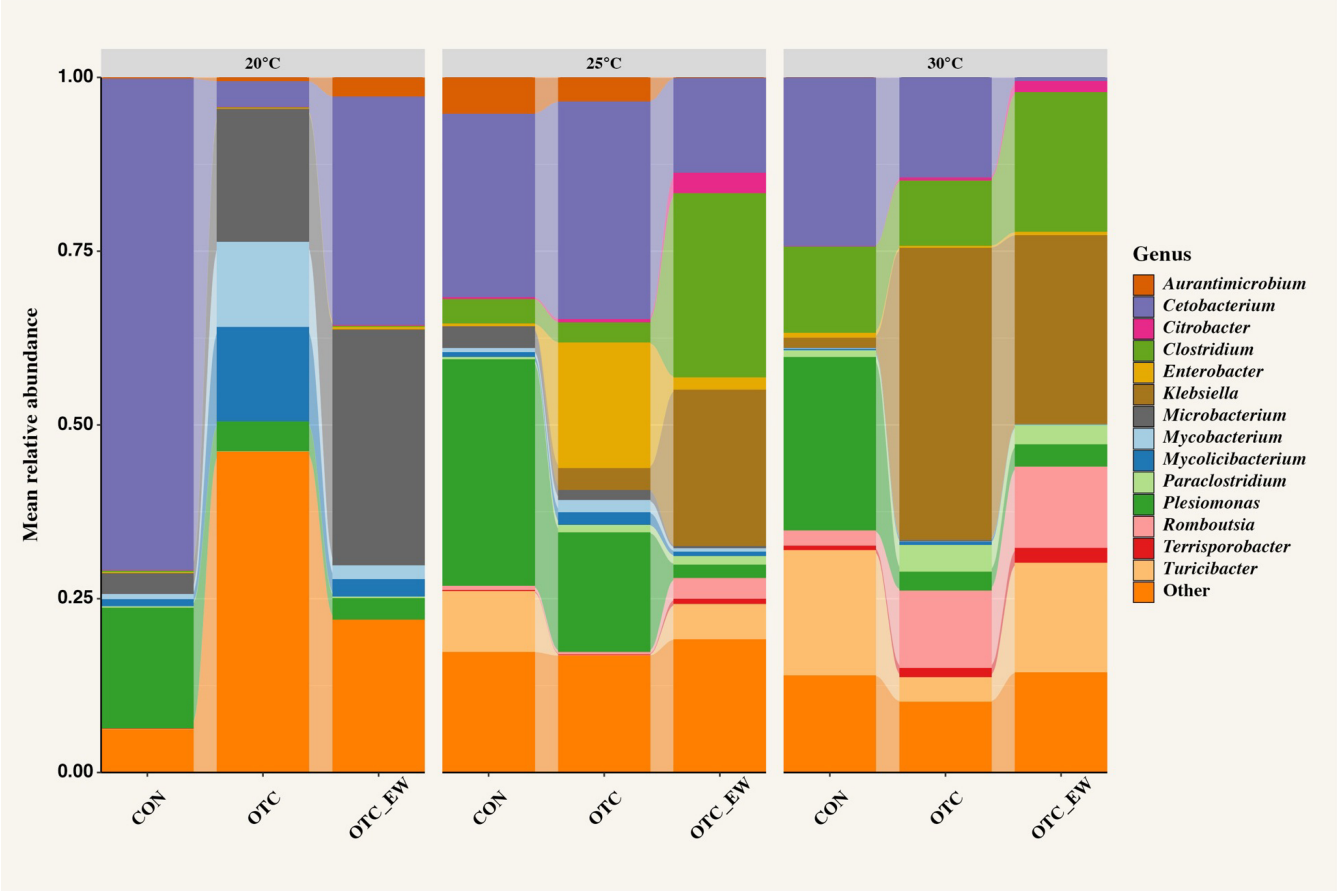
Relative abundance of dominant bacterial genera in the gut microbiome of channel catfish (*Ictalurus punctatus*) fingerlings under control (CON), oxytetracycline (OTC) treatment, and end-of-withdrawal (OTC_EW) conditions at 20°C, 25°C, and 30°C.

Relative abundance profiles revealed strong temperature- and treatment-dependent shifts in the gut microbiome of channel catfish fingerlings (Fig. 3). At 20°C, the gut microbiome was initially dominated by *Cetobacterium* (70.7%), followed by *Plesiomonas* (17.3%) and *Microbacterium* (3.0%). Immediately after OTC treatment, *Microbacterium* became the most relatively abundant genus (19.0%), followed by *Mycolicibacterium* (13.6%) and *Mycobacterium* (12.2%). In contrast, *Cetobacterium* and *Plesiomonas* decreased sharply to 3.7% and 4.3%, respectively. By the end of the withdrawal period, *Cetobacterium* partially recovered to 32.9%, regaining dominance, while *Microbacterium* continued to increase to 33.9%. *Plesiomonas* remained low at 3.1%.

At 25°C, control samples were dominated by *Plesiomonas* (32.6%), followed by *Cetobacterium* (26.4%) and *Turicibacter* (8.8%). Following OTC treatment, *Cetobacterium* remained the most relatively abundant genus (31.3%), although *Plesiomonas* declined to 17.2%. Notably, *Enterobacter* increased dramatically from 0.4% to 18.1% after treatment. By the end of the withdrawal period, *Clostridium* (26.5%) and *Klebsiella* (22.5%) emerged as the dominant taxa, with *Cetobacterium* reduced to 13.6%.

At 30°C, the microbial community was initially composed of *Plesiomonas* (24.9%), *Cetobacterium* (24.3%), and *Turicibacter* (18.0%), with relatively higher baseline levels of *Clostridium* and *Klebsiella* compared to the cooler temperatures. After OTC treatment, *Klebsiella* increased drastically from 1.4% to 42.1%, becoming the dominant genus, while *Cetobacterium* decreased to 14.43%. *Romboutsia* (11.3%) and *Clostridium* also rose in abundance, while *Turicibacter* declined. At the end of the withdrawal period, *Klebsiella* (27.2%), *Clostridium* (20.1%), and *Turicibacter* (15.8%) were the dominant taxa, with *Romboutsia* remaining stable at ~11.7%.

**Fig 4:**
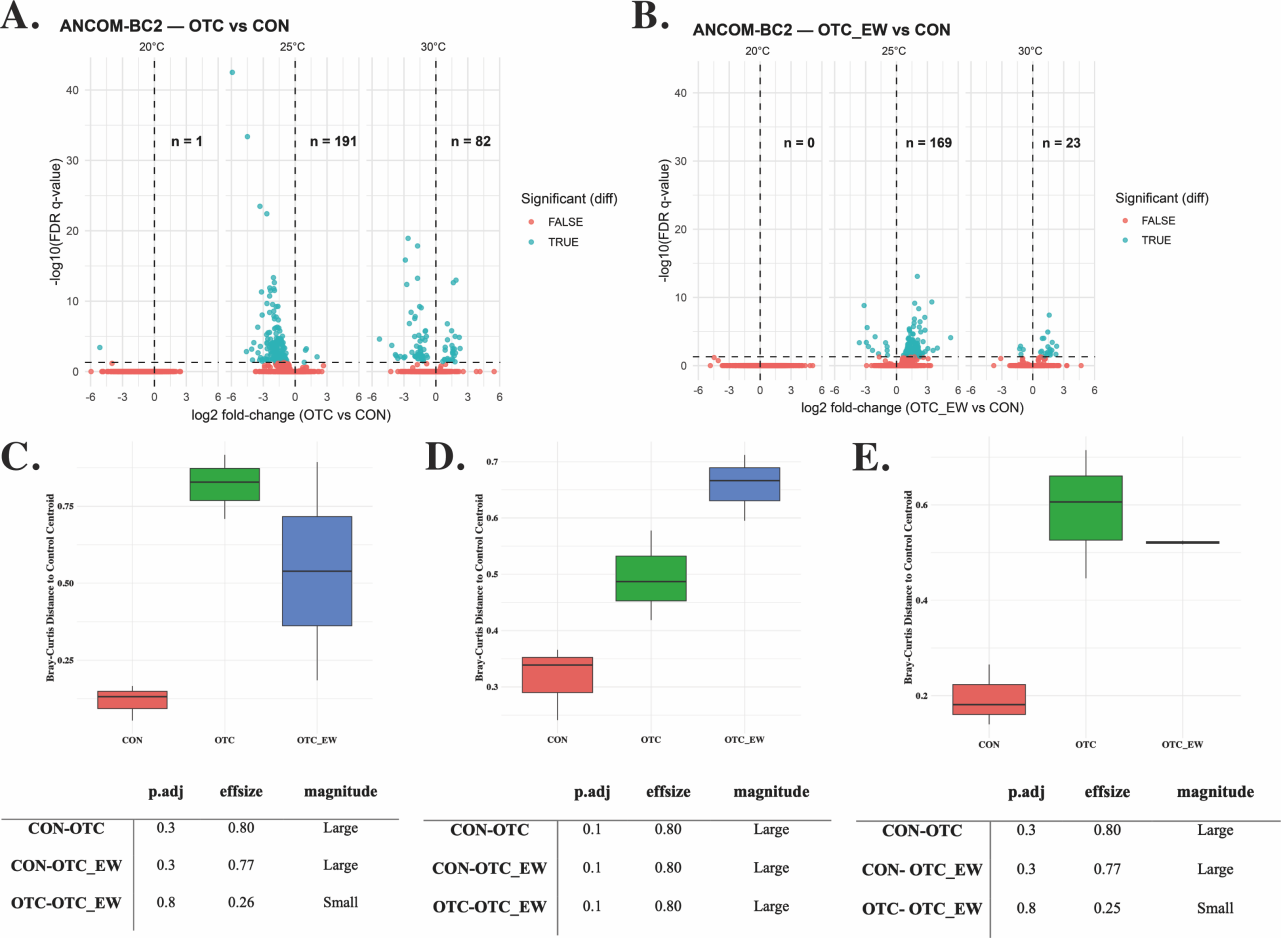
Differential abundance and community divergence in the gut microbiome of channel catfish (*Ictalurus punctatus*) following oxytetracycline (OTC) treatment across temperature conditions. (**A and B**) Volcano plots comparing OTC vs control (CON) (**A**) and end-of-withdrawal (OTC_EW) vs CON (**B**) at 20°C, 25°C, and 30°C. Blue points indicate significantly differentially abundant taxa (false-discovery rate [FDR] <0.05); red points indicate taxa without significant differences. (**C–E**) Assessment of community perturbation via Bray–Curtis distance to control centroids at 20°C (**C**), 25°C (**D**), and 30°C (**E**). Boxplots represent the dispersion of individual samples relative to the control mean, with larger distances reflecting more pronounced compositional shifts.

Differential abundance analysis was performed using ANCOM-BC2 to identify taxa responding to OTC treatment under each temperature condition (Fig. 4A and B). At 20°C, only one taxon showed a significant response to OTC treatment, and its abundance returned to baseline levels by the end of the withdrawal period. This suggests that the microbial community at the lower temperature was relatively stable and resistant to antibiotic perturbation. At higher temperatures, the impact was much greater. At 25°C, 191 taxa were significantly altered immediately after treatment, while 169 taxa remained significantly different from controls at the end of withdrawal (Fig. 4A and B). At 30°C, 82 taxa were significantly altered at the end of treatment, and 23 taxa remained different from controls at the end of withdrawal.

To further quantify these compositional shifts, we calculated the distance of each sample to the control group centroid based on Bray–Curtis dissimilarity during both the treatment and withdrawal stages (Fig. 4C and D). In all cases, treated samples were more distant from the control centroid than control replicates, regardless of temperature. Although these differences were not statistically significant (Kruskal–Wallis, *P* > 0.05), Wilcoxon rank-based effect size estimates indicated large effects (*r* ≥ 0.5). Together, these results suggest that OTC treatment induced dysbiosis and that the gut microbiome did not fully recover by the end of the withdrawal period, with temperature strongly influencing both the magnitude and persistence of community shifts.

### Temperature-dependent shifts in resistome abundance and composition under OTC treatment

**Fig 5:**
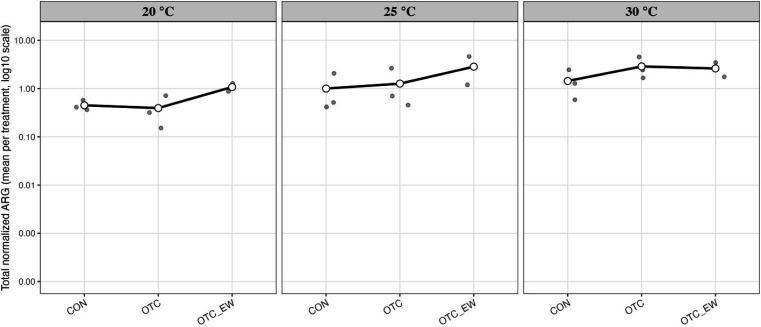
Impact of tetracycline on the relative abundance of antibiotic resistance genes (ARGs) in the gut microbiome of channel catfish (*Ictalurus punctatus*) fingerlings before, after, and at the end of the withdrawal period under different water temperatures.

OTC treatment overall increased total ARG abundance across all three temperatures, though the degree of change varied, and none reached statistical significance (Fig. 5). At 20°C, total ARG abundance decreased slightly immediately after treatment but rose above control levels by the end of the withdrawal period. At 25°C, baseline ARG abundance was higher than at 20°C and showed a continuous increase from treatment through withdrawal. At 30°C, total ARG abundance was higher at baseline than at both 20°C and 25°C, increased following treatment, but declined slightly by the end of withdrawal.

**Fig 6:**
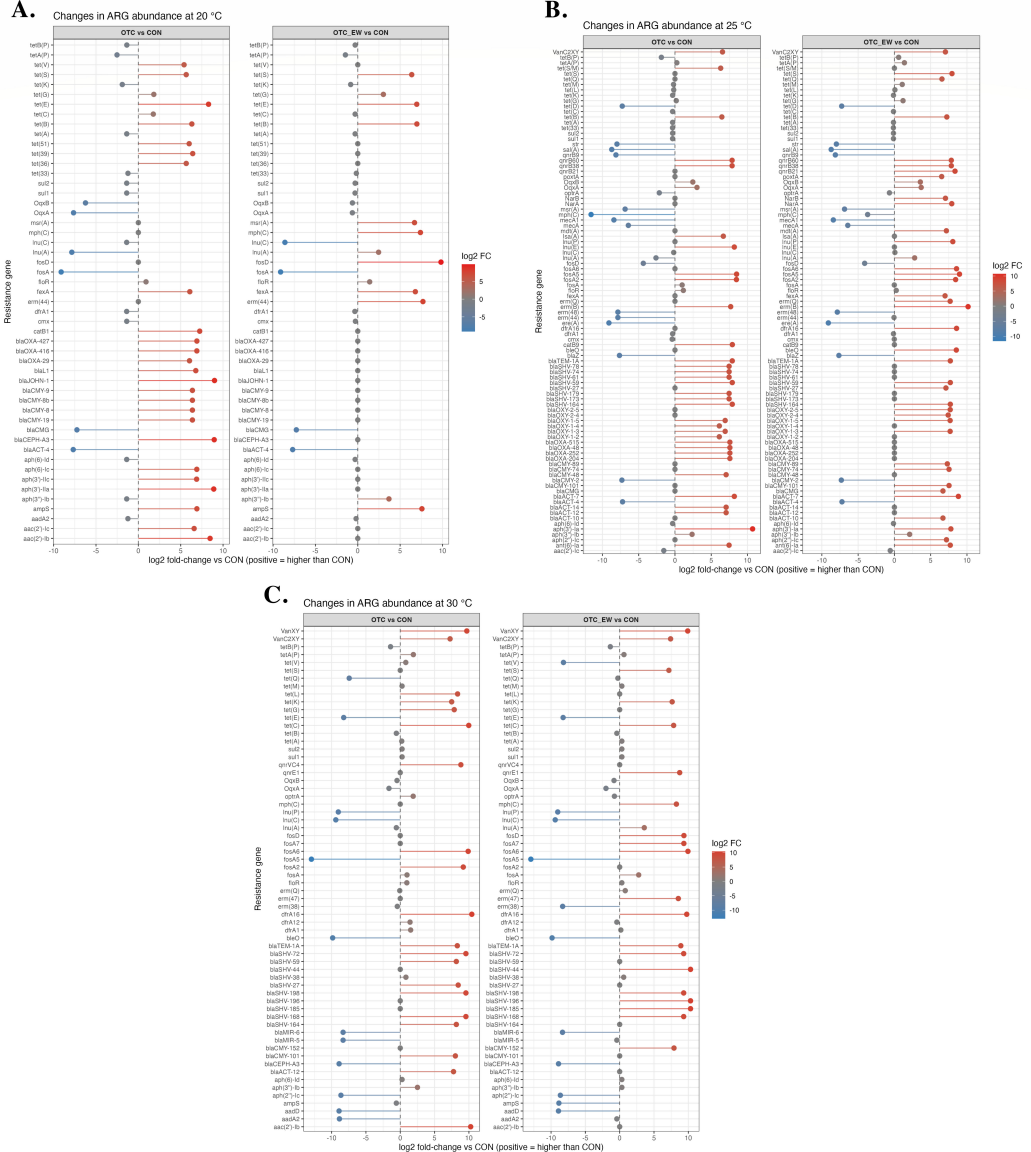
Enrichment of antibiotic resistance genes (ARGs) associated with different drug classes in the gut microbiome of channel catfish (*Ictalurus punctatus*) fingerlings before, after, and at the end of the tetracycline withdrawal period under (**A**) 20°C (**B**) 25°C, and (**C**) 30°C.

OTC exposure also produced distinct temperature-dependent shifts in ARG composition (Fig. 6). At 20°C, changes were relatively modest and transient, with enrichment primarily observed among tetracycline, β-lactam, and aminoglycoside resistance genes. By the end of withdrawal, only a limited set of genes, specifically *tet(S)*, *tet(E)*, *tet(B)*, *fexA*, and *ampS*, remained elevated. Interestingly, several macrolide–lincosamide–streptogramin resistance genes, including *msr(A)*, *mph(C)*, and *erm(44)*, emerged at this stage despite showing no enrichment immediately after treatment (Fig. 6A).

At 25°C, the resistome response was broader and more persistent (Fig. 6B). Multiple ARG families, including *tet*, *qnrB*, *fosA*, and *bla*, showed strong positive fold changes in response to OTC treatment. Many of these enrichments persisted, indicating sustained selection pressure at moderate temperature.

At 30°C, OTC also exerted a relatively strong effect compared to 20°C, driving widespread enrichment across diverse ARG classes (Fig. 6C). Enrichment was mainly observed in *van*, *fosA*, *tet*, and *bla* resistance genes. Importantly, many of these ARGs remained elevated after withdrawal.

### Taxonomic distribution of ARG-carrying contigs across temperatures

**Fig 7:**
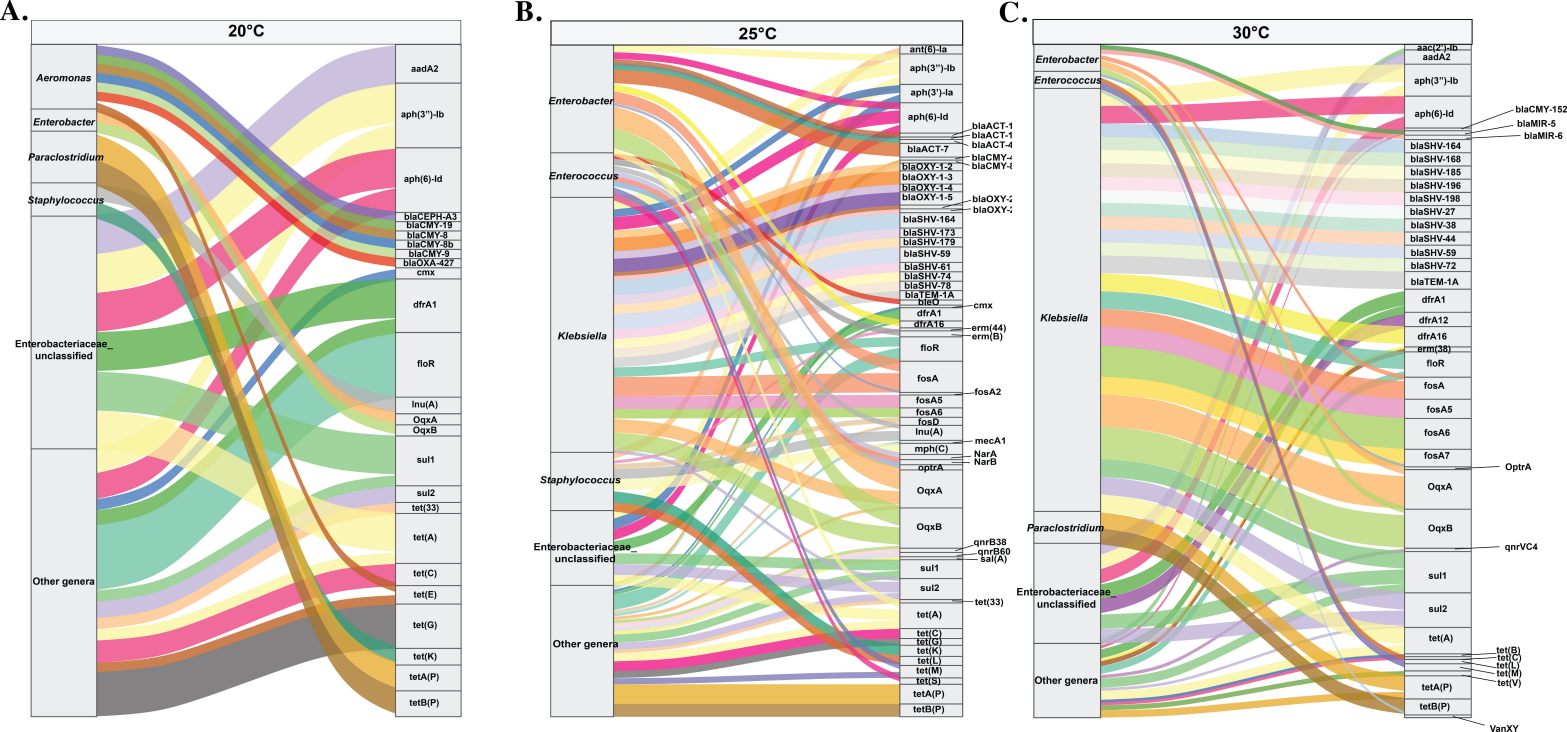
Antibiotic resistance gene (ARG)–host associations in the gut microbiome of channel catfish (*Ictalurus punctatus*) reared at 20°C (**A**), 25°C (**B**), and 30°C (**C**). Sankey diagram showing connections between the top five bacterial genera (ranked by ARG abundance within this temperature condition; genera below this cutoff were grouped as “other genera”) and all detected resistance genes. The left panel displays bacterial genera inferred from contig-level taxonomic classification, while the right panel shows the corresponding ARGs detected on those contigs. The height of each node (genus or gene) is proportional to the total number of ARGs assigned to that category. The width of each flow represents the number of contigs linking a given bacterial genus to a specific resistance gene. Flows representing fewer than five ARG counts were excluded for clarity.

To investigate the taxonomic distribution of ARG-carrying contigs, a genus-level assignment was performed on contigs harboring resistance genes at different temperatures, and the associations were visualized using a Sankey diagram (Fig. 7).

At 20°C, ARG-carrying contigs were taxonomically diffuse, with unclassified *Enterobacteriaceae* and “other genera” (a composite category aggregating all genera outside the top five ARG hosts by abundance in this condition) contributing the largest fractions (Fig. 7A). Unclassified genera within the *Enterobacteriaceae* primarily carried *sul1*, *tet(A)*, *dfrA1*, *aph(6)-Id*, *aadA2*, and *aph(3″)-Ib*. Most tetracycline resistance genes were associated with the other genera group, although *tet(A)* and *tet(B)* were specifically linked to *Paraclostridium*, and *tet(K)* was linked to *Staphylococcus*.

At 25°C, the ARG–host structure became more concentrated (Fig. 7B). *Enterobacter* and *Klebsiella* emerged as dominant ARG reservoirs, each connecting to multiple antibiotic resistance classes. *Klebsiella* exhibited strong linkages to distinct resistance determinants, including aminoglycoside resistance genes [e.g., *aph(3″)-Ia, aph(3″)-Ib*, and *aph(6)-Id*]*,* β-lactamase genes (such as *blaOXY* and multiple *blaSHV*/*blaACT* variants), and the multidrug efflux operon *oqxA/B*. Distinct associations were also predicted for tetracycline resistance: *Enterococcus* carried a diverse suite, including *tet(A)*, *tet(L)*, *tet(M)*, and *tet(S)*, whereas *Staphylococcus* was primarily associated *with tet(K)* and *tet(L)*.

At 30°C, ARG flows became highly concentrated among a few dominant taxa (Fig. 7C). *Klebsiella* was the most prominent reservoir, with strong associations with β-lactamases (e.g., *blaSHV* and *blaTEM* variants), *oqxA/B* efflux pumps, sulfonamide resistance genes (*sul1* and *sul2*), *fosA* (fosfomycin resistance), *dfrA* (trimethoprim resistance), and *tet(A)*. Tetracycline resistance genes were also frequently associated with *Paraclostridium*, *Enterococcus*, and other genera.

## DISCUSSION

Our previous study analyzed changes in the microbiome and resistome of channel catfish culture water under OTC stress across different temperatures (8). This current study serves as a follow-up, in which we further examined the microbiome and resistome of the channel catfish gut under similar conditions.

Temperature-dependent shifts in fish gut microbiota have been well documented (29, 55, 56). However, these responses appear to be highly context-dependent, varying according to host species, the magnitude and duration of thermal stress, and the specific gut compartment sampled. For instance, Zhang et al. (56) observed that raising water temperature from 24°C to 29°C did not significantly alter bacterial richness (OTUs), Shannon diversity, or Faith’s phylogenetic diversity in the gut microbiomes of common carp (*Cyprinus carpio*) or largemouth bass (*Micropterus salmoides*). In contrast, Wu et al. (29) reported that a modest 2°C increase (18°C–20°C) over 7 days significantly enhanced microbial richness and diversity in the intestinal mucosa of Nile tilapia (*Oreochromis niloticus*). In the present study, we confirmed baseline differences in the gut microbiome of channel catfish across 20°C, 25°C, and 30°C. These specific thermal points were selected to represent seasonal fluctuations in major catfish production regions and to span the optimal growth range (22°C–32°C) of *A. hydrophila*, the primary pathogen causing bacterial hemorrhagic septicemia and a common target for OTC treatment.

When OTC was applied, temperature-associated patterns in bacterial relative abundance were observed (Fig. 3). At 20°C, *Cetobacterium* showed lower relative abundance, while *Microbacterium*, *Mycobacterium*, and *Mycolicibacterium* showed higher relative abundance compared to the control. The ability of mycobacteria to tolerate tetracyclines is attributed to their complex cell envelope, which limits antibiotic permeability, as well as the expression of genes conferring specific resistance mechanisms (57, 58). Certain species, such as *Mycobacterium smegmatis* and *Mycobacterium tuberculosis*, display moderate tetracycline tolerance through TetV/Tap efflux pumps (59–61), while *Mycobacterium abscessus* is ~500-fold more resistant due to MabTetX, a monooxygenase that degrades tetracyclines (62). At higher temperatures (25°C and 30°C), the most notable shift was the marked increase in the relative abundance of *Klebsiella*, a major carrier of ARGs, after OTC treatment. Importantly, in a previous study, *Klebsiella* expansion was consistently observed in both the fish gut and the corresponding culture water at an elevated water temperature (8), underscoring its potential role in AMR dissemination in aquaculture.

Notably, the gut microbiome exhibited incomplete recovery by the end of the withdrawal period, particularly at 25°C and 30°C. A similar persistence of dysbiosis has been reported in rainbow trout (*Oncorhynchus mykiss*), where microbial richness and composition remained distinctly altered compared to controls 14 days after OTC treatment cessation (63). Likewise, in juvenile rainbow trout, dysbiosis persisted for at least 25 days after treatment (64). Resistome analysis revealed enrichment of β-lactamase (*bla*) genes under OTC exposure, consistent with co-selection rather than direct β-lactam pressure. Tetracycline resistance genes are frequently co-located with β-lactamase genes on plasmids, transposons, or integrons, meaning tetracycline exposure can indirectly select for β-lactamase genes by maintaining entire multidrug elements (65).

Despite the emergence of *Klebsiella* as a dominant taxon at 25°C and 30°C in both the fish gut and culture water (culture-water data from a parallel study [8]), important differences between the two environments were observed. The gut microbiome was dominated by *Cetobacterium*, *Plesiomonas*, *Microbacterium*, *Klebsiella*, and *Clostridium*, whereas culture water was enriched in *Aurantimicrobium*, *Mycobacterium*, *Flavobacterium*, *Aeromonas*, and *Klebsiella*. The response of the resistome to OTC exposure also diverged between environments (i.e., culture water vs fish gut). In culture water, OTC treatment significantly increased total ARG abundance and richness at 20°C and 30°C but had no significant effect at 25°C (8). In contrast, in the fish gut, the effects of OTC treatment on total ARG abundance across sampling phases (post-treatment and withdrawal) at all three temperatures were not statistically significant, likely due to limited sample size, but generally showed higher ARG levels by the end of withdrawal compared to the initial levels in controls. The impact of antibiotics was most pronounced at 25°C: total ARG abundance increased post-treatment and continued rising during withdrawal, with a wider diversity of genes remaining enriched compared to other temperatures.

The non-linear, temperature-dependent impact of OTC on the gut microbiome, along with discrepancies between gut and water responses, likely reflects fish physiology and antibiotic pharmacokinetics. In ectothermic animals, feeding, metabolism, and drug uptake are strongly temperature dependent (66). At ~20°C, channel catfish reduce feeding, often consuming medicated feed slowly or incompletely (67). Although efforts were made during our experiment to ensure complete feed consumption and consistent OTC dosing, prolonged feeding times likely allowed greater diffusion of OTC into the water, thereby reducing effective gut exposure. At ~30°C, by contrast, rapid metabolism and circulation accelerate drug clearance. For example, in Nile tilapia, clearance relative to bioavailability (CL/F; i.e., apparent oral clearance) and elimination half-life of amoxicillin were nearly doubled at 30°C compared to 25°C (68), suggesting shorter gut exposure and a reduced impact of OTC on the microbiome. At 25°C, which falls within the optimal feeding and metabolic range of warmwater fish, feed intake and drug absorption were likely maximized, while clearance rates remained moderate, resulting in the strongest dysbiosis. Additionally, antibiotic persistence in water may also contribute; the half-life of OTC is ~19 days at 22°C but extends to 41 days at 7°C (69).

These discrepancies between gut and water microbiomes are consistent with findings by Sadeghi et al. (70), who examined 334 freshwater fish across 17 species and 3 habitats. Their study showed that while the surrounding environment strongly influences host-associated microbiota (e.g., gut and skin), evolutionary relationships between fish and their microbiomes also play a significant role. Together, our results highlight the need to consider the fish gut and culture water as distinct yet interconnected environments when evaluating AMR emergence and dissemination in aquaculture systems. In addition, many previous studies investigating the impact of antibiotics on fish gut microbiomes have been conducted under a single temperature condition (63, 71–73). Our findings emphasize the importance of incorporating multiple temperatures, as baseline differences in gut microbiome composition strongly influence the response to antibiotics. Temperature not only shapes microbial community structure but also affects fish physiology, feeding behavior, and antibiotic pharmacokinetics, all of which can alter the extent and persistence of microbiome and resistome disruption.

Although producers cannot directly control pond water temperature, recognizing that certain temperatures intensify antibiotic impacts provides opportunities for risk mitigation. For example, proactive measures such as limiting unnecessary antibiotic courses, strengthening biosecurity, and incorporating microbiome recovery strategies (e.g., probiotics, optimized feeding regimes) could help buffer long-term impacts. More broadly, judicious antibiotic use, rotation of drugs when alternatives are available, and enhanced health monitoring at temperature “risk zones” should be considered as practical strategies to minimize AMR emergence.

The current study has several limitations. First, the experimental system is multifactorial: temperature, host physiology, and pond-water communities co-vary. Our design can mitigate but not eliminate confounding; thus, attributing OTC-driven gut microbiome changes solely to temperature should be interpreted cautiously. Second, gut inferences rely on DNA-based short-read metagenomics, which resolves community and ARG profiles but not absolute abundances or host responses. Future work would benefit from quantifying key targets (e.g., qPCR of elevated ARGs), tracking innate immune markers (lysozyme, complement), and including gut mucosal histology to incorporate host physiology. Third, the limited sample size likely constrained our ability to detect statistically significant differences, and time effects could not be formally evaluated because matched control fish were sequenced only at baseline (pre-treatment), whereas OTC fish were sequenced at later phases. Due to the small size of the fish and low digesta content, DNA yield and quality were suboptimal, leading to the exclusion of several samples. Future studies should prioritize optimizing sample collection and increasing biological replicates to improve statistical power and reproducibility.

## Data Availability

All sequencing data are available in the NCBI BioProject under accession number PRJNA1393111.

## SUPPLEMENTAL MATERIAL

Data S1 spectrum.04187-25-s0001.xlsb Water quality monitoring data.

ASM does not own the copyrights to Supplemental Material that may be linked to, or accessed through, an article. The authors have granted ASM a non-exclusive, world-wide license to publish the Supplemental Material files. Please contact the corresponding author directly for reuse.
